# A retrospective chromosome studies among Iranian infertile women: Report of 21 years

**Published:** 2013-04

**Authors:** Cyrus Azimi, Malihea Khaleghian, Farideh Farzanfar

**Affiliations:** *Genetics Group, Cancer Research Center, Cancer Institute of Iran, Tehran University of Medical Sciences, Tehran, Iran.*

**Keywords:** *Infertile women*, *Karyotyping*, *Chromosome abnormalities*

## Abstract

**Background: **The infertility is an important health problem, affecting about 15% of couples. The important role of genetic factors in pathogenesis of infertility is now increasingly recognized. The value of karyotyping women in the routine work-out of couples referred for sterility has long been recommended.

**Objective:** The aim of this study was to define the frequency of all chromosomal aberrations among women which referred to our department due to infertility during the 21-year period.

**Materials and Methods: **In this 21-year retrospective study, for the first time, we investigated 896 women which referred to our department due to infertility during 1986 to 2006. For chromosome analysis, heparinized peripheral blood samples were cultured, harvested and banded according to standard methods.

**Results:** Out of 896 patients, 710 patients (79.24%) had a normal karyotype, and 186 patients (20.76%) showed abnormal karyotype. Among the abnormal ones 48 patients (25.81%) showed Turner's syndrome (45,X), and 45 patients (24.19%) were sex reversal with 46,XY karyotype. The rest of 93 patients (50%) revealed a wide range of chromosome abnormalities.

**Conclusion:** Our results emphasized the importance of the standard cytogenetic methods in assessing the genetic characteristics of infertile females, which allows detecting a variety of somatic chromosome abnormalities, because some of these may interfere with the success of reproduction.

## Introduction

The infertility is an important health problem, affecting about 15% of couples (1). The important role of genetic factors in pathogenesis of infertility is now increasingly recognized (2). Chromosome aberrations may cause infertility in both men and women (3, 4). At the present time, although various laboratory tests are available to find out the cause of infertility, even at the molecular level, peripheral blood chromosome study remains the first choice in assessing the genetic characteristics of an infertile couple (5). The main cause of female infertility is amenorrhea, and it has been suggested that the prevalence of amenorrhea not due to pregnancy, lactation or menopause is around 3-4% (6, 7). 

Four conditions are accounted as main factors for amenorrhea including: polycystic ovary syndrome (PCOS), hypothalamic amenorrhea, hyperprolactinemia, and premature ovarian failure (POF). According to a collaborating investigation, single-gene defects are most likely to be found among patients with hypogonadotropic hypogonadism (8). Statistics shows that in the reproductive referral centers, the majority of visited cases were due to primary and secondary amnorrhea (9-11).

POF is characterized by absent menarche or premature depletion of ovarian follicles/ arrested folliculogenesis before the age of 40 (12, 13). This condition is distinguished by the presence of primary or secondary amenorrhea for at least 4 months, hypoestrogenism and elevated serum gonadotropin concentrations (14, 15). The diagnosis is confirmed by two blood tests at least 1 month apart to measure FSH (16, 17). POF incidence in patients with 46, XX karyotype was estimated one in 10,000 and 1,000 women by age 20 and 30, respectively. The familial form of POF is rare, representing 4-31% of all cases (18). 

Multiple causes of POF can be defined and result in follicle reduction and/or defects in the follicular development stimulus mechanism (13). Ovarian dysfunction can be secondary to autoimmune diseases, infections, chemotherapy and radiation treatment and metabolic diseases, but for most of the cases, the etiology is idiopathic and probably genetic (17, 19). The genetic basis to the disease is supported by the occurrence of families with several affected women (18, 20, 21). Regarding the genetic causes of POF, they can be chromosomal or caused by single genes (22). The X chromosome abnormalities represent 13% of the cases, and also there are many reports that suggested three X-linked and nine autosomal genes are involved in POF development (15, 23, 24). 

Turner syndrome is a common genetic disorder with an incidence of 1 in 2,500 females, and has been classically associated with a 45,X karyotype (15). Several X-chromosomal abnormalities have been identified in these patients. 45,X karyotype is found in 50-60% of the cases (25). The other cases are mosaics with a 45,X cell line accompanied by others with two or more X chromosomes or with structural anomalies. Such structural aberrations of the X chromosome (isochromosomes of the long arm, dicentric chromosomes, deletion of the short arm or ring chromosomes) are present in approximately 30% of the cases (26). Finally, around 5% are patients with structural abnormalities of the Y chromosome (isochromosomes of the long arm and dicentric chromosomes) and mosaics with at least one Y chromosome, whether complete or not (27).

Mendes *et al* suggested that about 25% of patients with Turner syndrome are mosaics and among them around 40% show Y-chromosome-specific sequences (28). Studies showed that the risk of gonadal tumors including gonadoblastoma and dysgerminoma is increased in Y-carrying patients with gonadal dysgenesis (29, 30). This confers clinical importance to the detection of the Y-chromosome mosaicism in Turner syndrome (31, 32). Turner patients are at risk for development of endocrine, autoimmune, and structural abnormalities. As many as 1.5% of the population with Turner syndrome may develop dissection of the ascending aorta. 5% of Turners may have abbreviated menstrual function before developing amenorrhea and hypergonadotropic hypogonadism. It is estimated that 1-2% of all patients may become pregnant. In nearly 80% of patients with a 45,X cell line, the X chromosome is of maternal origin. This suggests that the abnormality is usually a paternal meiotic or post-fertilization mitotic error. It is for this reason that it is not thought to be increased with advanced maternal age (33). 

POF is the most common cause of delayed spontaneous puberty in girls, and more of them had Turner syndrome than 46, XX and, more rarely, 46, XY-associated POF (9). Trisomy X is a sex chromosome aneuploidy and occurs in approximately 1 in 1,000 female births. Although 47,XXX karyotypes are the most frequent, mosaicism occurs in approximately 10% of cases and in many combinations such as 46,XX/ 47,XXX or 45,X/ 47,XXX or 47,XXX/ 48,XXXX or 45,X/ 46,XX/ 47,XXX (34). 

There have been numerous reports of women with trisomy X developing POF with endocrine findings of hypergonadotropic hypogonadism in the 19-40 year age group (35). Conversely, another study observed that 3% of patients with POF had trisomy X, and a high percentage of them were affected by autoimmune diseases (36). Chromosome studies have been recommended for women presenting with primary amenorrhea, premature menopause, and recurrent abortions (37-39). Jabbar has emphasized that management of these patients should be multidisciplinary and individualized according to the patient’s age and symptoms at presentation, and psychological counseling is also very important (40). 

The overall frequency of chromosome anomalies in patients attending a fertility clinic is around 2-3% for women (41, 42). Many researchers have reported different frequency of chromosome anomalies among the infertile women, including 2.01% in women undergoing intrauterine insemination (IUI), 1.8-2.5% among patients undergoing in-vitro fertilization (IVF), and 1.1-9.8% in female patients who were candidates for intracytoplasmic sperm injection (ICSI) (43-53). 

This retrospective cross sectional study reports the frequency of chromosome aberrations in the lymphocytes of 896 women which have been referred to our department, due to infertility, for a period of 21 years.

## Materials and methods

In this 21-year retrospective study, we investigated all the women referred to the Genetics Group, Cancer Institute of Iran, Tehran University of Medical Sciences, with a diagnosis of infertility during 1986-2006. This study was purely a laboratory (not clinical) investigation, and all the patients were referred from all over the country, by many obstetricians and gynecologists for cytogenetic studies. The referral clinicians claimed that their patients had a full medical history, general clinical examination and the other work-up including: sonography of uterine and ovaries/ hysterosalpingography, a full endocrine study, and semen analysis of the male partner. Therefore, in this study, inclusion and exclusion criteria were not applied.

Cytogenetic investigations were performed on peripheral blood cultured for 72 hours in the presence of phytohemagglutinin (PHA) (54, 55). According to the standard protocol, 5ml of heparinized blood was collected from every referred patient. Lymphocytes were cultured in culture media containing 100cc of RPMI-1640/ Ham’s F-10/ Ham’s F-12 (from GIBCO, UK) and or McCoy’s 5A (from SIGMA, Germany) as a base; 20cc Fetal Bovine Serum (from GIBCO/Invitrogen, UK); 2cc of Phytohemagglutinin (from GIBCO/Invitrogen, UK) as a mitogenic agent; and 1cc of Penicillin/Streptomycin (from GIBCO/ Invitrogen, UK). The samples were incubated for 72 hours at 37^o^C. The metaphases were arrested with adding 0.1% Colchicine/ Colcemid (from GIBCO/Invitrogen, UK). High resolution was performed by using 1% Thymidine (from SIGMA, Germany) for obtaining prometaphase chromosome preparations.

5ml of hypotonic solution (KCl 5.6 g/lit) was added and mixed well and incubated for 15 minutes at 37^o^C. The cells were fixed with three washes of fixative consisted of 3:1, methanol:acetic acid (from MERK, Germany). 

Chromosome staining and banding techniques were as described by de Grouchy and Turleau, and Benn and Perle (56, 57). G-bandig was carried out for each sample. In all cases, for routine chromosome analysis, 30 Giemsa-banded cells were studied; 20-25 cells were counted and 5-10 cells were analyzed, using two separate blood tubes from each patient (58). If there was any indication for mosaicism, in addition to the mentioned procedure, 200 metaphases were scanned again from that two separate blood tubes. 

For every abnormal karyotype, except G-banding, other techniques were used. Q-banding was performed for confirmation of chromosome Y abnormalities, sex reversal cases, studies on acrocentric chromosomes (59). High resolution banding was carried out for assurance of structural abnormalities (60). Due to lack of Automated Karyotyping Systems or any softwares, all the analyses were carried out manually, under the light microscope, by highly expert technicians.

All karyotypes were interpreted in accordance with the recommendation of the International System for Human Cytogenetic Nomenclature (ISCN) (61, 62).

## Results

Cytogenetic analysis was performed on 896 Iranian infertile women for a period of 21 years. 710 patients (79.24%) had a normal karyotype, and 186 patients (20.76%) showed abnormal karyotype (Table I). Among the abnormal ones 48 patients (25.81%) showed Turner's syndrome with 45,X karyotype, which was the most frequent anomaly in our investigation, and 45 patients (24.19%) were sex reversal with 46,XY karyotype. 

The rest of 93 patients (50%) revealed a wide range of chromosome abnormalities which is shown in Table II. Different mosaics of Turner’s syndrome were seen in 26 patients (13.98%). 27 persons (14.52%) were observed to have isochromosome X, 11 cases (5.91%) with deletion of chromosome X, and 4 patients (2.15%) with ring chromosome X.

**Table I:** Chromosome analysis of all referred infertile women

**Karyotype**	**Number**	**Percentage of total**
46,XY(sex reversal)	45	5.03
45,X	48	5.37
mos 45,X/46,XX	20	2.24
mos 45,X/46,XY	4	0.45
45,X,inv(1)(p22p34)	1	0.11
mos 47,XXX/46,XX	3	0.33
mos 47,XXX[25]/45,X[20]	1	0.11
47,X,+fis(X)(p10),+fis(X)(q10)	1	0.11
46,X,i(X)(q10)	11	1.24
mos 46,X,i(X)(q10)/45,X	14	1.57
mos 46,X,i(X)(q10)[33]/45,X[21]/47,XXY[10]/46,XX[6]	1	0.11
mos 46,X,i(X)(q10)[31]/47,XXX[19]/45,X[12]/46,XX[8]	1	0.11
46,X,del(X)(q24)	2	0.22
46,X,del(X)(q11)	3	0.33
46,X,del(X)(q13)	1	0.11
46,X,del(X)(q21)	1	0.11
mos 46,X,del(X)(q13q22)[27]/45,X[18]	2	0.22
mos 46,X,del(X)(p11)[25]/45,X[20]	1	0.11
mos 46,X,del(X)(p11)[36]/46,XX[9]	1	0.11
mos 46,X,del(Y)(q11)[25]/45,X[20]	1	0.11
mos 46,X,r(X)(p22.1q26)[22]/45,X[18]	1	0.11
mos 46,X,r(X)(p22.2q27.3)[23]/45,X[17]	1	0.11
mos 46,X,r(X)(p11.4q13.3)[34]/45,X[16]	1	0.11
mos 46,X,r(X)(p21.1q13)[38]/45,X[62]	1	0.11
46,X,psu idic(X)(p11)	1	0.11
46,X,psu idic(X)(q24)	1	0.11
mos 46,X,psu idic(X)(p11)[38]/45,X[32]	1	0.11
mos 46,X,psu idic(X)(q22)[51]/45,X[9]	1	0.11
46,X,t(X;2)(q22;q23)	1	0.11
46,X,t(X;19)(q22;q13.3)	2	0.22
47,XX,+mar	2	0.22
mos 46,X,+mar/45,X	6	0.67
46,XX,inv(9)(p11q13)	4	0.45
46,XX,9qh+	1	0.11
Sub-total	186	20.76
46,XX	710	79.24
Total	896	100

p: short arm of chromosome.          q: long arm of chromosome.           mos: mosaic.           i: isochromosome.          del: deletion.

r: ring chromosome.                         t: translocation.                              inv: inversion.          h: heterochomatin.         mar: marker chromosome.

psu idic: pseudoisodicentric.          fis: fission.

**Table II:** Classification of chromosome abnormalities among all referred infertile women

**Chromosome Abnormalities**		**Number**	**%**
Sex reversal		45	24.19
Numerical abnormalities	45 chromosomes	73	39.25
Number: 78 (41.94%)	47 chromosomes	5	2.69
	Isochromosomes X	27	14.52
	Deletions X	12	6.45
Structural abnormalities	Ring chromosomes X	4	2.15
Number: 63 (33.87%)	Pseudoisodicentric X	3	1.61
	Translocations X and autosomes	4	2.15
	Markers	8	4.30
	Inversions of chromosome 9	5	2.69
Total		186	100

**Figure 1:**
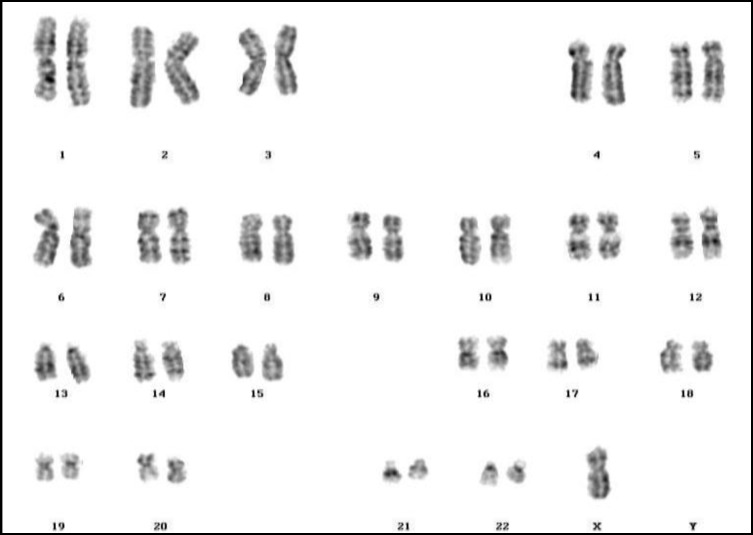
45, X

**Figure 2:**
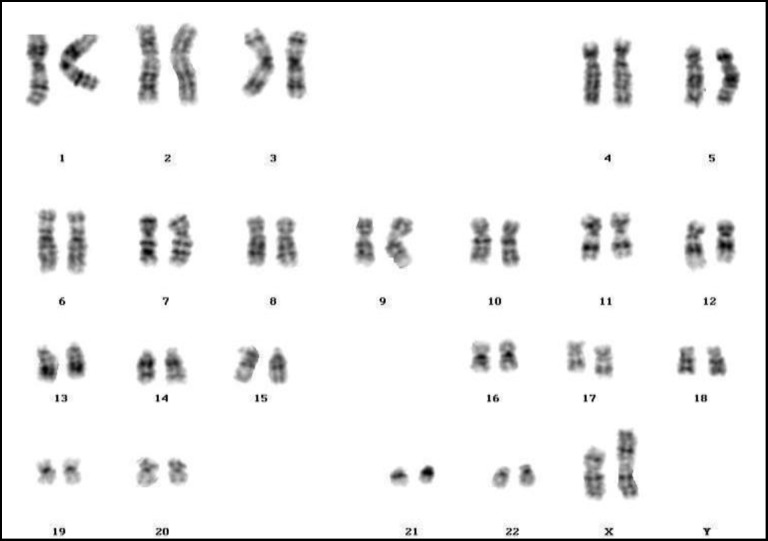
46,X,i(X)(q10

**Figure 3:**
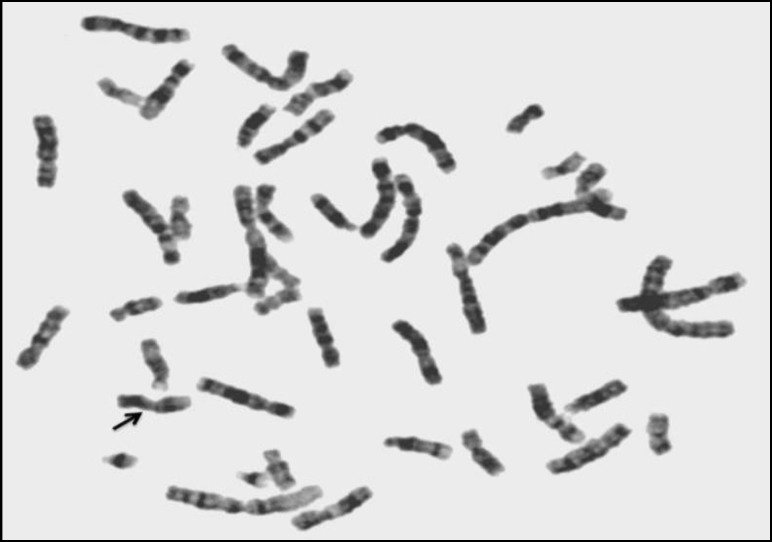
46,X,del(X)(q24)

**Figure 4:**
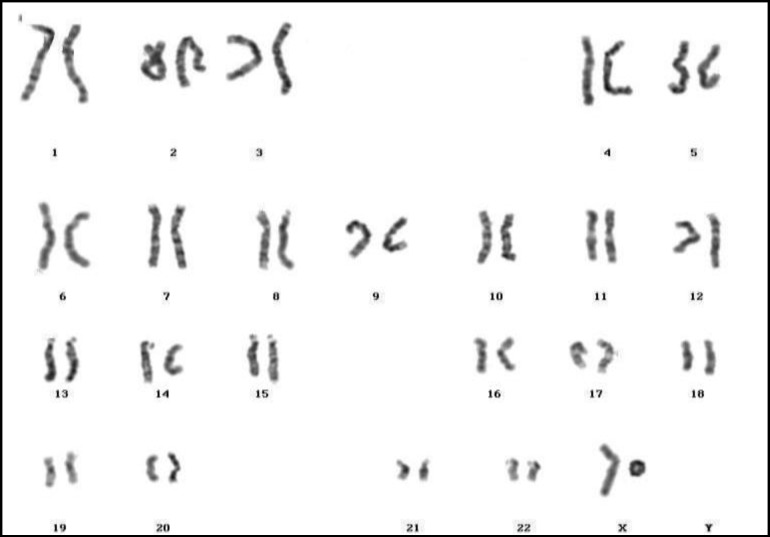
46,X,r(X)(p22.2q27.3)

**Figure 5:**
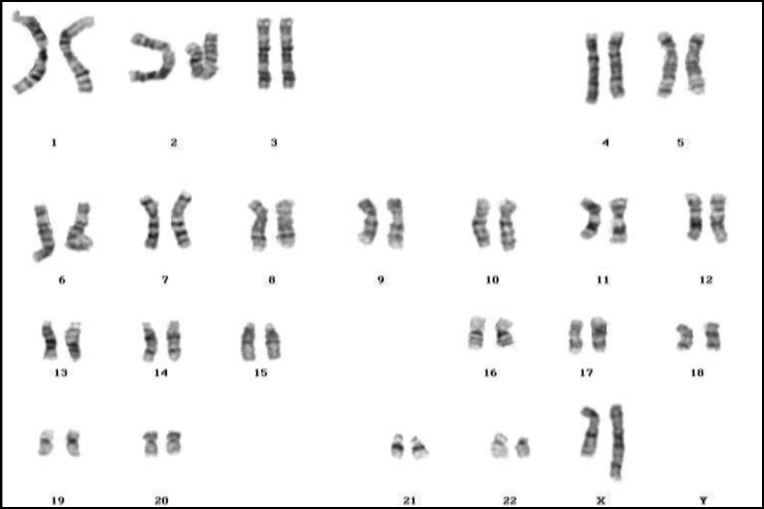
46,X,psu idic(X)(q22)

## Discussion

Although the most common causes of female infertility are; ovulation disorders, blocked fallopian tubes, polycystic ovary syndrome (PCOS) and endometriosis, but chromosome abnormalities can also be one of the important causes. In the present study, 48 cases (25.81%) with Turner’s syndrome (Figure I), 26 patients (13.98 %) with three various forms of mosaic Turner’s syndrome were the most prevalent abnormalities as were seen in the previous literatures (50, 63, 64). There was also one case of Turner syndrome with inversion of chromosome 1 (45,X,inv(1) (p22p34).

Trisomy X or triple X syndrome is characterized by the presence of an additional X chromosome in each of a female’s cells. Although females with this condition may be taller than average, this chromosomal change typically causes no unusual physical features. Some females with triple X syndrome are not able to conceive children. Several authors have shown female infertility among patients with trisomy X or various forms of its mosaics (50, 51, 63, 64). We had also four cases (2.15%) with mosaics of 47,XXX syndrome.

Some researchers have reported female infertility among patients with isochromosome X, deletion of chromosome X, ring chromosome X, pseudo-isodicentric X, and with marker chromosome (44, 52, 62-68). Similarly, in this investigation we found eleven cases (5.91%) with 46,X,i(X)(q10) (Figure II), and sixteen patients (8.60%) with various mosaics of isochromosome X; seven patients (3.76%) with 46,X,del(X) (Figure III), and four cases (2.15%) with mosaic del(X); four patients (2.15%) with various mosaics of 46,X,r(X) (Figure IV); two cases (1.08%) with pseudoisodicentric X, and two cases (1.08%) with mosaic pseudoisodicentric X (Figure V); and eight patients (4.30%) with marker chromosome.

According to Van der Ven *et al*, Clementini *et al*, Papanikolaou *et al*, and review by Chantot-Bastaraud *et al* gonosomal mosaics including 45,X cell lines, and also various inversions, reciprocal and Robertsonian translocations are commonly found in infertile females (5, 51, 68, 69). Most of these aberrations could also be detected in our study, such as two cases (1.08%) with t(X;19), and one patient (o.54%) with t (X;2). 

In the present study we found 45 females (24.19%) with sex reversal and 46,XY karyotype. Swyer syndrome, or XY gonadal dysgenesis, is a type of hypogonadism in a chromatin negative person whose karyotype is 46,XY. The patients appear to be normal females who do not, however, develop secondary sexual characteristics at puberty, do not menstruate, and have streak gonads. Affected sisters were reported by Cohen and Shaw, and twins by Frasier *et al *(70, 71). Sternberg *et al* observed 3 cases, each in a different sibship of a family connected through normal females (72). A high incidence of neoplasia (gonadoblastomas and germinomas) in streak gonads of patients with the XY karyotype was shown by Taylor *et al* (73).

Polymorphic variants, particularly involving the heterochromatic region of chromosomes 1, 9, 16 and the nucleolar organizing region of acrocentric chromosomes, are known to occur in 2.7% of the general population. However, much higher frequencies (12.2-38%) have been reported in infertile individuals (74, 75). Studies by Sahin *et al*; Minocherhomji *et al* and Purandare *et al* also showed that heteromorphisms shown by paracentric long-arm regions of chromosomes 1, 9 and 16 were associated with infertility (76-78). In this investigation we found one patient (0.54%) with 46,XX,9qh+.

Inversion of chromosome 9 is commonly seen in normal humans and the frequency has been reported to be 1 to 3% in the general population, and some authors account the inv as a normal variant (9, 67, 79-81). Capkova *et al* investigated chromosomal abnormalities in couples with reproductive disorders, and showed that structural aberrations, including inversion 9, were more frequent among infertile couples (82). Some authors reported inv among women with recurrent abortions, suggesting that these inversions can have a role in the causation of infertility, especially in cases with de novo inversions. Khaleghian and Azimi's suggestion further confirmed this. We also had four cases (2.15%) with 46,XX,inv (p11q13) karyotpe among our sample (9, 83-85).

The observed overall frequency of such chromosome abnormalities varies between different investigations. For instance, Mau-Holzmann reported abnormal karyotypes of 8.7% among 4327 female patients (86). Another review was carried out by de Braekeleer *et al* on 8390 women, and showed the mean rate of abnormal karyotypes of 4.2% (87). Rajangam *et al* found 11.5% of females with bad obstetric history such as: spontaneous abortions, live births with congenital malformations, and stillbirths have had a chromosomal abnormality as a genetic cause (63). In contrast, the frequency of aberrations in some other studies varies from 0.87 to 2.34% for female patients (68, 88-90).

In accordance with other investigations (86, 87) a considerable number of our female patients revealed a phenomenon known as low-level sex chromosome mosaicism, i.e. the occurrence of a few metaphases with hypoploidy and/or hyperploidy of sex chromosomes. Although the exact role of low level sex chromosome mosaicism in ovarian function has not yet been clarified, the association of low-level 45,X mosaicism with POF could somehow help us understand this role (91, 92). 

Since there is a high rate of X chromosome loss in patients with POF, varying degrees of the disease is observed which could be attributed to chromosome mosaicism. Moreover, it was suggested that premature menopause could occur in women with X chromosome mosaicism (65, 91-93). Usually, the loss of an X chromosome is more frequent than its gain as also ascertained in the present study (86). de Braekeleer *et al* concluded that the presence of two 45,X cells or more reflects true mosaicism (87).

Many researchers have reported a lower frequency of chromosome anomalies among the infertile women. In the present investigation, we found 20.76% of our referred female patients with chromosome abnormalities, which it was higher than other reports. The reason is that our patients were highly selected group. Our patients have been passed through many filters, including they have been examined and tested by obstetrician/gynecologist, endocrinologist, and if the diagnosis of them was chromosome abnormality, then they were referred to us. Our data is in agreement with the results of Devroey *et al* which found that up to 26% of women with non-surgical primary ovarian failure show an abnormal karyotype (94). 

They suggested that the overall frequency of chromosomal aberrations is strongly influenced by gynecological and andrological causes. Baronchelli *et al* emphasized the importance of X chromosome in the etiology of POF and highlighted the potential role of low-level sex chromosome mosaicism in ovarian aging that may lead to a premature onset of menopause (95). Therefore, along with Gekas *et al*, Papanikolaou *et al*, Romero Tovar *et al *and Rosenbusch our studies confirm that, routine peripheral blood chromosome analysis remains the first choice in assessing the genetic characteristics of infertile women (5, 64, 96, 97). 

## Conclusion

Our results emphasized the importance of the standard cytogenetic methods in assessing the genetic characteristics of infertile females, which allows detecting a variety of somatic chromosome abnormalities, because some of these may interfere with the success of reproduction.
